# Public Procurement Practices for Cereal Products in Polish Educational Institutions: Analysis and Implications for Nutrition Policy

**DOI:** 10.3390/nu16172880

**Published:** 2024-08-28

**Authors:** Katarzyna Brukało, Aleksandra Kołodziejczyk, Justyna Nowak, Oskar Kowalski

**Affiliations:** 1 Department of Health Policy Faculty of Public Health in Bytom, Medical University of Silesia, 40-055 Katowice, Poland; 2 Student Scientific Circle, Department of Metabolic Disease Prevention, Faculty of Public Health in Bytom, Medical University of Silesia, 40-055 Katowice, Poland; s79887@365.sum.edu.pl; 3 Department of Metabolic Disease Prevention, Faculty of Public Health in Bytom, Medical University of Silesia, 40-055 Katowice, Poland; justyna.nowak@sum.edu.pl; 4 Department of Human Nutrition, Faculty of Public Health in Bytom, Medical University of Silesia, 40-055 Katowice, Poland; okowalski@sum.edu.pl

**Keywords:** public food procurement, cereal products, schools, kindergartens, nutrition quality, sustainability, sensorial characteristics, nutrition policy, food policy

## Abstract

Public procurement of food is crucial for ensuring proper nutrition and the provision of high-quality products in public institutions like schools and kindergartens. It should be seen as an investment in health promotion, particularly for young children. Notably, when no quality criteria are specified, the cheapest and often lowest-quality products are typically selected. This study analyzed 1126 public procurement orders processed by schools and kindergartens in Poland between November 2022 and March 2023, with a focus on cereal products and their derivatives. Of these orders, 197 met the inclusion criteria, yielding a total of 5084 cereal products for detailed analysis. The study assessed the quantities ordered and the quality characteristics specified in the procurement documents. The results revealed that the most commonly described criteria pertained to product composition, especially typical characteristics and the absence of additives. Sensorial characteristics such as consistency and color were also frequently specified, while sustainable public procurement criteria were mentioned the least, indicating their marginal importance in current procurement practices. This underscores the critical importance of establishing minimum standards for describing cereal products in terms of sensorial characteristics, composition, and sustainability. Such standards are essential for improving the quality of grain products supplied to public institutions and ensuring that these institutions actively contribute to promoting healthy eating habits among children.

## 1. Introduction

The formation of eating habits occurs during childhood and is subsequently reinforced throughout adulthood, resulting in the establishment of long-term behavioral patterns [1,2]. Inappropriate dietary habits in youth may result in the onset of diet-related illnesses in adulthood, including type 2 diabetes and hypertension [2,3,4].

Cereal products are crucial in the diets of children and adolescents because they provide essential nutrients that support growth and development. They are a primary source of carbohydrates, which supply the energy needed for daily activities and physical growth. Excessive consumption of simple carbohydrates, such as those found in white bread and sweetened cereals, can lead to several health issues. These rapidly absorbed sugars cause spikes in blood glucose levels, which can contribute to insulin resistance and increase the risk of developing type 2 diabetes. Additionally, frequent consumption of simple carbohydrates is associated with an increased risk of obesity, as these foods often lead to overeating due to their low satiety levels. Whole grains, in particular, are rich in dietary fiber, which aids digestion, and contain key vitamins like B vitamins, which are important for brain function, and minerals such as iron and magnesium, which are essential for building strong bones and supporting the immune system. Regular consumption of high-quality whole grain cereals helps establish healthy eating habits early on and reduces the risk of developing chronic conditions like obesity, type 2 diabetes, and heart disease later in life [1,2,3,4].

The development of eating habits in young people is influenced by a number of factors, including the home environment and the social environment [2,3]. Modifying eating behaviors and controlling body weight in children represents a significant challenge. However, nutritional practices employed in educational institutions offer a promising avenue for intervention [1,3]. A healthy diet is defined as a nutritional model that ensures the adequate supply of essential nutrients and provides an amount of energy that meets the metabolic needs of the individual while avoiding both energy deficiencies and excess [3].

Polish food legislation is primarily based on national and EU regulations, with the Food and Nutrition Safety Act of 2006 representing the principal legislative instrument in this field [5]. In Poland, the public procurement system is regulated by the Public Procurement Law [6]. The Public Procurement Bulletin, as regulated in the aforementioned Act, serves to centralize all public procurements in Poland. Following a period of three months, the documentation related to individual proceedings is deleted, leaving only general data [6]. In the context of public procurement, educational institutions are advised to provide precise specifications regarding the desired characteristics of the procured products, despite the absence of explicit regulatory guidance. The prevailing regulatory framework pertaining to nutrition primarily concerns the products that are available for purchase and the principles governing the composition of menus [5]. 

In accordance with the Education Law, kindergartens and primary schools are obliged to provide students with a daily hot meal, which can be purchased on a voluntary basis. The entire menu should encompass a diverse range of food groups [7]. A balanced diet should comprise a variety of food items from different food groups, including grains or potatoes, vegetables or fruit, milk or dairy products, meat, fish, eggs, nuts, legumes, and fats [5]. Cereal products constitute a fundamental element of a healthy diet, providing energy, fiber, vitamins, and minerals [8,9,10]. In accordance with Polish legislation, educational institutions are required to provide at least one portion of cereal products on a daily basis for breakfast, lunch, and dinner [5]. It is recommended that whole grain products be the predominant component, including groats, whole grain pasta, brown rice, whole meal and graham bread, and cereal flakes, such as oatmeal [11]. A diet rich in whole grain products has been linked to a number of beneficial effects, including positive impacts on the nervous system and digestive system and maintenance of appropriate body weight [8,9,11,12]. 

The incorporation of whole grain cereal products into the daily diet of children and adolescents is associated with enhanced overall health and well-being, while also reducing the risk of developing diseases related to poor nutrition [8,9,10,11]. It is of particular importance to pay close attention to the quality and source of food products consumed by young people, as an excess of products containing simple sugars, such as sweet breakfast cereals, may increase the risk of developing type 2 diabetes, and an excess of salt in the diet may lead to the development of arterial hypertension [13,14]. It is therefore of great importance to select cereal products appropriately for the diets of young people. In the case of educational institutions, the selection criteria should not be limited mainly to the cost aspect [2,3,4].

In Poland, the procurement of food by public institutions is governed by the general public procurement law [6]. This legislation does not include specific provisions related to nutrition policy. The Public Procurement Office indicates that procuring entities can consider various environmental factors, such as buying organic, seasonal, or sustainably produced food, but these are merely recommendations [15].

To enhance and simplify the public procurement process, Poland has utilized the E-Procurement Platform since 2022. All entities engaged in public procurement are required to log in to this platform and upload all relevant documents. The platform includes the Public Procurement Bulletin, which facilitates searching for and accessing information about specific procurements. According to the platform’s guidelines, all documents associated with a public procurement are deleted within three months of the procurement’s completion.

The aim of the analysis was to examine public procurement practices for cereal products in schools and kindergartens and to evaluate the quality criteria used in the selection of these products. The analysis sought to understand the preferences of the procurers and to identify the most and least frequently used criteria for ordered products across different cereal categories. Given that cereal products are a staple of the daily diet according to the food pyramid, their procurement and quality are particularly important [11]. To our knowledge, this is the first analysis of public procurement of cereal products.

## 2. Materials and Methods

To collect information on public tenders for food products ordered by kindergartens and primary schools for the year 2023, data were extracted from the Public Procurement Bulletin over the period from 15 November 2022 to 15 March 2023. The search was based on CPV codes (15000000-8—food, beverages, tobacco, and related products, specifically including grain products).

A comprehensive database was established, encompassing 1126 public procurement entries related to these CPV codes processed by the specified educational institutions. The selection criteria for the analysis were restricted to initial procurements (excluding any procedures repeated due to invalidity) and required the availability of complete documentation to ensure the reliability of the analysis.

The final dataset comprised 197 public contracts meeting the following criteria:

- Processed by an educational institution (kindergarten, primary school, or school-preschool complex);
- Representing the initial procurement (fulfilling the complete commodity needs of the institutions);
- Featuring complete documentation (including order descriptions, assortment and price forms, draft contract provisions, information on allocated budgets, and procedure settlements).

**Figure 1:**
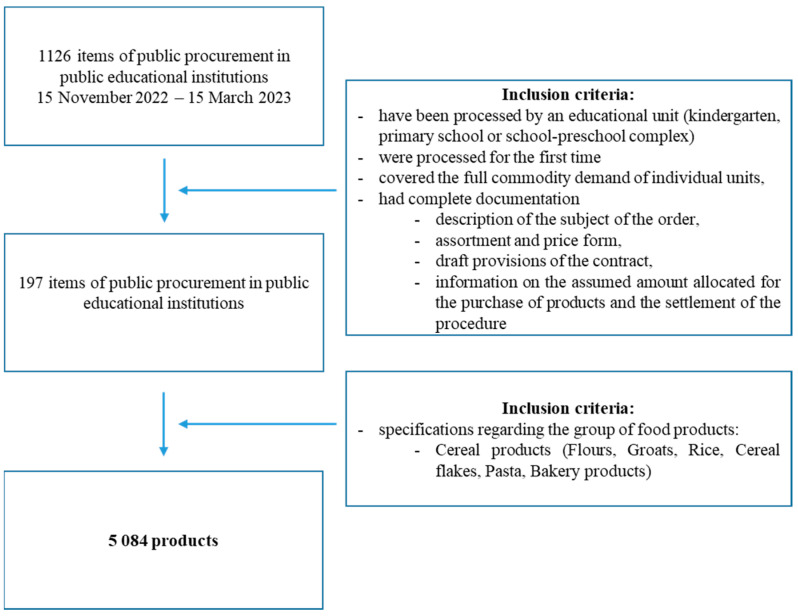
Characteristics of inclusion criteria.

From this dataset, a detailed database of ordered grain products was compiled, listing 5084 items (Figure 1).

A quantitative analysis was conducted for each ordered product, determining the number of packages, units, or kilograms procured. Following this, a qualitative review was performed based on the quality descriptions provided in the public procurement orders for all 5084 products. This review allowed for the identification of the criteria most frequently specified by the procuring entities, as follows:

- Product composition:
  ○ Without additives;
  ○ Content of sugar/sweeteners;
  ○ Sodium/salt content;
  ○ Typical product characteristics;
  ○ Absence of contamination;
  ○ Gluten-free status.
- Sensorial characteristics:
  ○ Taste;
  ○ Smell;
  ○ Color;
  ○ Appearance/consistency.
- Sustainable procurement:
  ○ Locality of products;
  ○ Organic products;
  ○ Packaging type (cartonboard/glass);
  ○ Large packaging volume (>1 kg).

This study represents a detailed and comprehensive analysis of public procurement practices for cereal products and provides a novel insight into procurement criteria and quality standards within the educational sector in Poland. An analogous analysis was conducted by the authors for dairy products.

## 3. Results

The group of cereal products contained 5084 products, which were divided into subgroups. The applicable breakdown is shown in the following table (Table 1):

### 3.1. Flours

Flours were ordered in 182 procurement proceedings. The most frequently chosen products were wheat flour (253 products, 61,397.73 kg) and potato flour (163 products, 6800 kg). The least commonly ordered products were millet flour (1 product) and buckwheat flour (6 products). (Table 2).

Among the wheat flours, the most frequently ordered were cake flour—type 450 (120 products, 47.43%) and luxury flour—type 500 (59 products, 23.32%). Conversely, the least frequently ordered were flour type 2000 (8 products, 3.16%) and type 1850 (2 products, 0.79%). For 18 products (7.11%), it was specified that the flour should be whole grain.

In terms of sensorial characteristics, the most commonly required attributes were a specific, uniform white color (32 products, 6.06%) and a dry, free-flowing consistency without clumps (18 products, 3.41%). For three products (0.58%), it was also noted that the flour should have a specific smell consistent with the product.

Significant emphasis was placed on the “purity” of the flour. It was highlighted that the flour should be free from organic contaminants (36 products, 6.95%) and inorganic contaminants (31 products, 5.98%), as well as pests and their residues (32 products, 6.18%).

Regarding product composition criteria, it was stressed that the flour should be free of added flavors (7 products, 1.35%) and should not contain added sugar and should have low salt/sodium content (5 products, 0.97%). Additionally, 24 products (4.63%) specified a particular composition—100% of the specified grain.

### 3.2. Groats

Various types of groats were included in 182 procurement proceedings. A total of 78,120.14 kg of different groats were ordered across all proceedings. The most frequently ordered groat was pearl barley, with 314 products and a total of 31,821.67 kg. Other commonly ordered groats included buckwheat (166 products; 14,442 kg), bulgur (108 products; 12,119.67 kg), and semolina (178 products; 7636.80 kg). The least frequently ordered groats were oat groats (4 products; 120 kg), pearl groats (4 products; 357 kg), and rice groats (4 products; 310 kg). A detailed breakdown is provided in Table 3.

Among the barley groats, which are popular in Poland, the most preferred were pearl barley (75 products, 23.58%), country barley (17 products, 5.35%), and country barley (39 products, 12.26%). The most frequently ordered type was medium-grain barley groats (85 products, 26.73%), followed by coarse-grain barley groats (26 products, 8.18%), and the least ordered was fine-grain barley groats (5 products, 1.57%).

Among buckwheat groats, roasted buckwheat was ordered more often (44 products, 26.51% of buckwheat groats) compared to unroasted buckwheat (18 products, 10.84% of buckwheat groats). For semolina, instant semolina was ordered 42 times (23.6%).

Regarding bulgur, it was specified for 17 products (15.74%) that it should be made from durum wheat, and it was required to be made from cracked grains (6 products, 5.56%), dried grains (4 products, 3.70%), or cooked grains (4 products, 3.70%).

Sensorial criteria were considered only for 127 products (12.49%). These criteria most frequently concerned the consistency of the groats in terms of its dryness (127 products, 12.49%) and the integrity of the grains (41 products, 4.03%). Additionally, it was required that the groats be free from foreign odors (13 products, 1.28%) and that their color be appropriate for the type of groats (25 products, 2.46%).

Composition criteria were specified for 57 products (0.56%). It was most often noted that the groats should contain 100% of the appropriate grain (57 products, 0.56%), and that the groats should be free from added sugar (7 products, 0.69%) and sweeteners (7 products, 0.69%) and have low salt/sodium content (7 products, 0.69%), as well as being free from artificial additives (4 products, 0.39%) and preservatives (3 products, 0.29%).

### 3.3. Rice

The most frequently ordered rice by schools and kindergartens was white rice (142 products, 18,365 kg) and parboiled rice (85 products, 18,202 kg). The least frequently ordered types were Arborio rice (4 products, 211 kg), wild rice (4 products, 90 kg), and puffed rice (3 products, 75 kg).

The dataset also includes a general category “rice” because the type of rice was not specified for 47 products (7541 kg). Details are provided in Table 4.

Sensorial criteria were specified for 59 products (15.76%). These primarily concerned the consistency (51 products, 13.64%), which should be dry, free-flowing, non-sticky, and contain less than 10% broken grains and/or flour. The ordered rice should also have the desired color—white or slightly translucent (16 products, 4.28%)—and a characteristic aroma (9 products, 2.41%).

The buyers required that the rice be free from contaminants (46 products, 12.30%), particularly organic contaminants (17 products, 4.55%) and mineral contaminants (6 products, 1.60%), and that it be free from pests and their residues (34 products, 9.09%).

Additionally, it was specified that the product should not contain preservatives (7 products, 0.80%), colorants (5 products, 1.34%), or monosodium glutamate (5 products, 1.34%).

### 3.4. Cereal Flakes

The most popular items among the orders were corn flakes (148 products, 5272.07 kg) and oat flakes (124 products, 3506.6 kg). Flavored flakes (86 products, 3506.6 kg) and muesli (37 products, 985.7 kg) were also frequently included in the orders. Detailed data are presented in Table 5.

Among flavored flakes, chocolate-flavored flakes (41 products, 47.67%) and honey-flavored flakes (15 products, 17.44%) were predominant. Additionally, a significant portion of all ordered flakes were instant flakes (75 products, 13.23%).

For 44 products (7.76%), the buyers specified a particular expected composition. Quality requirements primarily focused on the addition and content of sugar in the product. For 50 products (8.82%), it was expected that the products be free of added sugar (mainly concerning corn flakes (29 products, 19.59%) and flavored flakes (10 products, 11.63%)). For 20 products (3.53%), the sugar content should not exceed 15 g per 100 g of the finished product, and for 4 products (0.71%), the sugar content was to be reduced (though the exact amount was not specified). Additionally, it was expected that the flakes have reduced sodium/salt content (21 products, 3.70%) or that this content be limited to 1 g per 100 g of the finished product (4 products, 0.71%).

Moreover, it was noted that the flakes should be gluten-free (12 products, 2.12%), but this requirement mainly applied to corn and rice flakes (only one case referred to oat flakes).

Among the required sensorial characteristics, the most frequently emphasized was that the consistency should be dry and free-flowing (73 products, 12.87%), with specific taste (21 products, 3.70%) and aroma (22 products, 3.88%), and correct color (12 products, 2.12%). Additionally, it was expected that the products be free from contaminants (50 products, 8.82%).

### 3.5. Pasta

The most frequently ordered pasta by Polish schools and kindergartens was wheat pasta (772 products, 62.06%) and durum wheat pasta (303 products, 24.36%). The least frequently purchased types of pasta were stuffed pasta (1 product, 6 kg), vegetable pasta (3 products, 62.5 kg), and spelt pasta (4 products, 158 kg) (see Table 6).

It is worth noting that the orders also included gluten-free pasta (30 products, 2.41%) and vegetable pasta (3 products, 0.24%).

For 128 products (10.29%), the desired specific composition was specified in the order (100% flour, eggs, water). For 117 products (9.41%), the expected egg content was detailed. This was expressed either as a percentage of the mass of the finished product (9 products, 0.72%)—ranging from 5.5% to 18%—or as the number of eggs added (108 products, 8.68%)—ranging from 1 to 8, with the most common being 5 (55 products, 4.42%).

It was also emphasized that the pasta should be free of any additional substances (116 products, 9.32%), particularly preservatives (15 products, 1.21%).

Among the sensorial characteristics, consistency was once again the most crucial aspect for buyers—the pasta in the package should not be broken, the surface should not be rough, and it should be elastic and non-sticky when cooked (192 products, 15.43%). The expected color of the pasta should be specific (usually pastel or cream) without specks and discolorations (38 products, 3.05%). The taste (7 products, 0.56%) and aroma (19 products, 1.52%) should be natural and characteristic.

### 3.6. Bakery Products

In schools and kindergartens, breads were ordered more frequently (761 products). For 58 products (4.28%), the buyers did not specify the type of bread or rolls ordered. Additionally, it should be clarified that the estimation of order quantities in kilograms only includes orders with a specified weight in kilograms (1092 products). For 262 products, only the product description and the number of pieces were provided, without specifying their weight. Detailed information is presented in Table 7.

Regarding rolls, the most frequently selected types are wheat rolls (256 products, 43.17%) and graham rolls (98 products, 16.53%). For bread, the most commonly chosen are mixed wheat–rye (173 products, 22.73%) and rye bread (86 products, 11.30%).

Flavored breads and rolls are also included in the selection. These mostly consist of sunflower seed bread, but occasionally include pumpkin, nut, or carrot bread.

Baked goods should be free from dents, mechanical damage, or mold (179 products, 13.22%). Additionally, they should be free from burning (40 products, 2.95%), produced within a maximum of 12 h from baking (31 products, 2.29%), and should not be made from frozen dough (24 products, 0.18%) or semi-finished products (8 products, 0.59%). For 111 products (8.20%), the requirement was simply that the bread should be fresh.

A specific ingredient composition was required for 223 products (16.47%), and for 48 products (3.55%), a specific type of flour was indicated. Additionally, bread should be made with sourdough (125 products, 9.23%) or natural sourdough (44 products, 3.25%). For 250 products (18.46%), the inclusion of yeast in the composition was specified. The use of the following was not allowed in the ingredients: leavening agents (173 products, 12.77%), preservatives (140 products, 10.34%), dough conditioners (154 products, 11.37%), artificial colorants (37 products, 2.73%), and flavor enhancers (36 products, 2.66%).

Regarding sensorial characteristics, the most common requirements were for the crust to be rough (88 products, 6.50%), shiny (52 products, 3.84%), and integrated with the crumb (108 products, 7.98%). The crumb should be dry and have uniform porosity (32 products, 2.36%). The color of the bread should be brown or golden (60 products, 4.43%), and its aroma should be aromatic and typical for the type of bread (61 products, 4.51%).

### 3.7. Sustainability Criteria

Sustainability criteria in public procurement of cereal products for schools and kindergartens were applied sporadically.

Local sourcing was specified exclusively for 4 products, specifically potato starch, where it was indicated that the starch should be produced from potatoes of Polish origin.

Organic standards were noted for 6 bakery products, where it was specified that the use of palm oil or L-cysteine derived from hair should be prohibited.

Environmentally friendly packaging, specifically paper packaging, is also uncommon. Traditionally, products such as cereals and rice are packaged in plastic bags. Paper bags are most frequently used for flour (58 products). Additionally, bulk packaging is generally not preferred by buyers, with the exception of bread, where delivery in plastic baskets is specified.

### 3.8. Collective Sheet

Among the classified criteria, those related to the product’s composition or its characteristic features are the most frequently used by procurers, while sustainability criteria are the least commonly applied.

It is important to emphasize, however, that the majority of products lack specified quality criteria, sensory attributes, or sustainability considerations. Detailed information is provided in Table 8.

## 4. Discussion

Public procurement of cereal products for schools and kindergartens is a key element in ensuring adequate nutrition standards in educational institutions. An analysis of public procurement results conducted in Poland in 2023 highlights the diversity of ordered products and the quality criteria aimed at ensuring proper nutrition standards. It should be noted that when price is the main (and sometimes the only) real criterion for selection, this choice will always come at the expense of product quality. These results provide valuable information that can contribute to a better understanding of purchasing behaviors in educational institutions and can serve as a basis for further research and optimization of procurement processes.

Primarily, the catalog of ordered cereal products features traditional items (e.g., pearl barley, which is present in the traditional Polish diet), universal products (such as wheat flour/not only used for baking but also for preparing various flour-based side dishes for main courses, such as dumplings, pancakes/and white rice, known for their versatility and common use in various recipes), and affordable and readily available options (like wheat bread) and those that are quick to prepare (such as muesli or sweetened breakfast cereals). This reflects the fact that these products are refined flour products, the consumption of which should be limited, especially among children and adolescents. This is particularly concerning in a country where one in three preschoolers is overweight, and children are gaining weight faster than in other European countries [16]. 

It should be emphasized that the orders also include products rich in complex carbohydrates, such as buckwheat, bulgur, and whole grain bread, which suggests an interest in healthier food choices among a limited portion of the institutions. The implementation of quality criteria in public procurement in Poland could significantly improve the quality of children’s nutrition while also educating the younger generation about healthy eating habits. Previous international interventions in schools aimed at increasing the consumption of whole grain products have resulted in better overall physical and mental well-being of students [17,18,19].

The analysis of public procurement procedures also revealed that the criteria employed (when used) are often applied in a chaotic and inadequate manner.

Firstly, some orders are missing essential details, such as the specific type of rice, groats, or bread, and the weight of portions or packaging. This lack of information is problematic because it allows the supplier to interpret the order, potentially leading to the provision of the cheapest, lower-quality product.

Moreover, even when orders include specified criteria, they are often applied inadequately. For example, a requirement for corn or rice flakes to be gluten-free is unnecessary and irrelevant, given that these products are naturally gluten-free. This stems from the fact that Poland lacks a standardized set of criteria for procurement officers to use when preparing purchase procedures. Additionally, there are no regulations ensuring that procurement officers receive education in human nutrition, which is essential for competently preparing these procedures. As a result, in our study, the majority of institutions made suboptimal choices, frequently procuring refined flour products rather than healthier alternatives. Therefore, it is crucial to establish standardized criteria that procurement officers could use when ordering cereal products for schools and kindergartens.

In developing procurement criteria, incorporating sustainability aspects such as local sourcing, organic farming, and eco-friendly packaging is essential. Local sourcing supports regional economies, reduces transportation emissions, and often provides fresher, higher-nutrient products. Organic farming avoids synthetic pesticides and fertilizers, enhancing soil health and biodiversity. Eco-friendly packaging uses recyclable or biodegradable materials, helping to minimize waste and reduce environmental impact [20]. Despite these benefits, the analysis shows that such sustainability criteria are currently underutilized.

Although Poland has the “Regulation of Minister of Health” [5], it only addresses menu composition principles (cereal products served once a day) and does not regulate the standards of the products used. The experiences of other countries could provide inspiration for such standardized criteria. Many countries have implemented specific public procurement criteria for educational institutions. For example, Denmark and Sweden employ stringent guidelines concerning organic and local food products [21,22]. In the United Kingdom, the “School Food Standards” program sets specific requirements regarding nutrient content and promotes the consumption of whole grain cereal products [23]. 

International examples show that implementing rigorous school nutrition standards yields tangible health benefits. Adopting similar public procurement criteria in Poland could significantly enhance the quality of children’s nutrition while also educating the younger generation about healthy eating habits. However, adapting these practices requires support from the government/local authorities and the engagement of all stakeholders, including suppliers, parents, and teachers [24].

To address the identified issues and improve public procurement practices, the following recommendations are proposed:

- Establish clear, standardized criteria. Develop and implement standardized criteria for public procurement of grain products. These criteria should encompass composition requirements, sensorial characteristics, and sustainability aspects.
- Enhance training for procurement officers. Introduce mandatory training programs for procurement officers to ensure they have the necessary knowledge in nutrition and quality standards.
- Promote sustainable practices. Incorporate sustainability criteria, such as local sourcing, organic farming, and eco-friendly packaging, into procurement policies.

Adopting these recommendations could significantly improve the quality of school meals (not only in Poland) and foster more informed and sustainable procurement practices in the public sector [25].

Eating habits form during childhood and have a long-term impact on health, including the risk of developing diet-related diseases such as type 2 diabetes or hypertension. Modifying eating habits and controlling body weight in children is a challenge, but nutritional practices used in educational institutions offer promising prospects. Public food procurements for schools and kindergartens are one of the main expenses from an economic perspective, and from the perspective of children and adolescents, one of the main meals during the day. From a public health perspective, it should be seen as an investment in health promotion. Nutritional products with a good composition will allow for the preparation of nutritious meals, influencing the nutritional status of children and adolescents.

## 5. Conclusions

The research objective of analyzing public procurement for cereal products in schools and kindergartens was met. The study successfully identified key trends and areas for improvement in the procurement practices of these institutions. An analysis of public procurement for cereal products in schools and kindergartens revealed that the most frequently ordered products were refined flour products, such as wheat flour, while millet and buckwheat flour were the least ordered. However, positive changes are noticeable in the procurement of durum wheat pasta and oatmeal. Despite these improvements, educational institutions still purchase sweetened cereals (e.g., muesli or honey flakes) and mainly wheat bread. There is nothing inherently wrong with purchasing these types of products, but their quality is crucial. This quality should be clearly defined in public procurement criteria, which, as shown, are significantly lacking. 

Further details from the study highlighted several key points, as follows:

- Many institutions are starting to introduce more whole grain options, which are beneficial for children’s health due to their higher fiber content and essential nutrients. Durum wheat pasta, which is a healthier alternative to regular pasta, and oatmeal, which is rich in fiber and beneficial for heart health, are becoming more common in school menus.
- Despite the shift towards healthier options, there is still a significant presence of sweetened cereals in school and kindergarten menus. These include products like muesli and honey flakes, which, while popular, are often high in added sugars. This highlights a need for stricter guidelines on sugar content in procured cereal products.
- Wheat bread remains a staple in many educational institutions. While wheat bread can be part of a healthy diet, there is a need for more whole grain options, such as whole wheat or multigrain bread, which offer additional health benefits compared to refined wheat bread.
- The study found that there is a significant lack of focus on sourcing products locally and choosing organic options. This absence means that potential benefits for the local economy and sustainable agricultural practices are not being realized. Incorporating these practices could have long-term benefits for both the environment and public health.
- Increasing the knowledge and competence of public procurement officers is crucial. These officers need to be well-versed in nutritional guidelines and the importance of high-quality ingredients to make informed decisions that benefit children’s health. This often remains a challenge for local governments, which act as the purchasers.

It is particularly important to establish and/or systematize standards for cereal products that children and adolescents consume regularly. These standards should concern composition, sensorial features, and sustainable public procurement. Clear and stringent public procurement criteria are essential to ensure that the nutritional quality of the foods served to children meets high standards. 

## Figures and Tables

**Table 1:** Breakdown of cereal products ordered for schools and kindergartens in 2023—number of products and volume.

Type of Cereal Products	Number of Products (n)	% of All Products	Order Volume [kg]	% of Order Volume
Flours	528	10.39%	70,243.73	11.12%
Groats	1017	20.00%	78,120.14	12.37%
Rice	374	7.36%	49,960	0.79%
Cereal Flakes	567	11.15%	16,810.95	2.66%
Pasta	1244	24.47%	104,910.1	16.61%
Bakery Products	1354	26.63%	356,457.28	56.44%
Grand Total	5084	100.00%	631,538.2	100.00%

**Table 2:** Type and quantity of flour ordered for schools and kindergartens in 2023 (based on public food procurements).

Type of Flour	Quantity [kg]	% of Total	Number of Orders	% of Orders
Wheat Flour	61,397.73	87.41%	253	47.92%
Potato Flour	6800	9.68%	163	30.87%
Corn Flour	848	1.21%	45	8.52%
Spelt Flour	442	0.63%	19	3.60%
Rice Flour	312	0.44%	14	2.65%
Rye Flour	295	0.42%	18	3.41%
Gluten-Free Flour	96	0.14%	9	1.70%
Buckwheat Flour	43	0.06%	6	1.14%
Millet Flour	10	0.01%	1	0.19%
Total	70,243.73	100.00%	528	100.00%

**Table 3:** Type and quantity of groats ordered for schools and kindergartens in 2023 (based on public food procurements).

Type of Groat	Quantity [kg]	% of Total	Number of Orders	% of Orders
Other Groats	90	0.12%	2	0.20%
Oat Groats	120	0.15%	4	0.39%
Quinoa	310	0.40%	4	0.39%
Spelt Groats	777	0.99%	9	0.88%
Corn Groats	1067	1.37%	36	3.54%
Millet Groats	4363	5.58%	100	9.83%
Couscous	5016	6.42%	92	9.05%
Semolina	7636.80	9.78%	178	17.50%
Bulgur	12,119.67	15.51%	108	10.62%
Buckwheat Groats	14,442	18.49%	166	16.32%
Pearl Barley	32,178.67	41.19%	318	31.27%
Total	78,120.14	100.00%	1017	100.00%

**Table 4:** Type and quantity of rice ordered for schools and kindergartens in 2023 (based on public food procurements).

Type of Rice	Quantity [kg]	% of Total	Number of Orders	% of Orders
White Rice	18,365	36.76%	142	37.97%
Parboiled Rice	18,202	36.43%	85	22.73%
Rice	7541	15.09%	47	12.57%
Brown Rice	3398	6.80%	60	16.04%
Basmati Rice	976	1.95%	11	2.94%
Jasmine Rice	635	1.27%	10	2.67%
Whole Grain Rice	467	0.93%	9	2.41%
Arborio Rice	211	0.42%	4	1.07%
Wild Rice	90	0.18%	3	0.80%
Puffed Rice	75	0.15%	3	0.80%
Total	49,960	100.00%	374	100.00%

**Table 5:** Type and quantity of cereal flakes ordered for schools and kindergartens in 2023.

Type of Flake	Quantity [kg]	% of Total	Number of Orders	% of Orders
Corn Flakes	5272.07	31.36%	148	26.10%
Oat Flakes	3506.6	20.86%	124	21.87%
Flavored Flakes	2632.68	15.66%	86	15.17%
Rice Flakes	1462.7	8.70%	67	11.82%
Muesli	1150.01	6.84%	30	5.29%
Barley Flakes	985.7	5.86%	37	6.53%
Millet Flakes	871.1	5.18%	36	6.35%
Spelt Flakes	499.7	2.97%	20	3.53%
Rye Flakes	294.4	1.75%	10	1.76%
Buckwheat Flakes	84.5	0.50%	4	0.71%
Granola	51.5	0.31%	5	0.88%
Total	16,810.95	100.00%	567	100.00%

**Table 6:** Type and quantity of pasta ordered for schools and kindergartens in 2023 (based on public food procurements).

Type of Pasta	Quantity [kg]	% of Total	Number of Orders	% of Orders
Wheat Pasta	53,831.67	51.31%	772	62.06%
Durum Wheat Pasta	42,426.55	40.44%	303	24.36%
Whole Grain Pasta	6913.6	6.59%	95	7.64%
Rye Pasta	774.25	0.74%	18	1.45%
Gluten-Free Pasta	283.25	0.27%	30	2.41%
Rice Pasta	239.5	0.23%	12	0.96%
Rye Flour Pasta	214.8	0.20%	6	0.48%
Spelt Pasta	158	0.15%	4	0.32%
Vegetable Pasta	62.5	0.06%	3	0.24%
Stuffed Pasta	6	0.01%	1	0.08%
Total	104,910.1	100.00%	1244	100.00%

**Table 7:** Type and quantity of bakery products ordered for schools and kindergartens in 2023 (based on public food procurements).

Bakery Products—Type	Quantity [kg]	% of Total	Number of Orders	% of Total Orders
Rolls				
Wheat	125,459	85.60%	256	43.17%
Graham	6581.05	4.49%	98	16.53%
Unspecified	4622.18	3.15%	44	7.42%
Multigrain	3452	2.36%	46	7.76%
Flavored	3018.74	2.06%	69	11.64%
Wheat–Rye	1329.80	0.91%	15	2.53%
Corn	719.20	0.49%	15	2.53%
Rye	711.35	0.49%	15	2.53%
Yeast	319.50	0.22%	11	1.85%
Soy	142.92	0.10%	3	0.51%
Rye	94.03	0.06%	4	0.67%
Spelt	71.15	0.05%	10	1.69%
Barley	15	0.01%	1	0.17%
Oat–Spelt	15	0.01%	1	0.17%
Gluten-Free	11.40	0.01%	4	0.67%
Millet	9	0.01%	1	0.17%
Total Rolls	146,571.30	100.00%	593	100.00%
Breads				
Wheat–Rye	75,416	35.93%	173	22.73%
Wheat	32,073	15.28%	76	9.99%
Rye	20,896	9.96%	86	11.30%
Whole Wheat	18,767	8.94%	68	8.94%
Graham	13,938	6.64%	64	8.41%
Multigrain	13,171	6.28%	73	9.59%
Flavored	12,378	5.90%	58	7.62%
Spelt	7100	3.38%	41	5.39%
Rye–Whole Wheat	5605	2.67%	26	3.42%
Unspecified	3445	1.64%	14	1.84%
Whole Grain	2569	1.22%	18	2.37%
Corn	1210	0.58%	12	1.58%
Toast	803	0.38%	19	2.50%
Buckwheat	640	0.30%	6	0.79%
Oat	530	0.25%	4	0.53%
Millet	500	0.24%	1	0.13%
Soy	330	0.16%	3	0.39%
Flaxseed	225	0.11%	2	0.26%
Bran	200	0.10%	1	0.13%
Potato	50	0.02%	2	0.26%
Gluten-Free	40	0.02%	13	1.71%
Crispy		0.00%	1	0.13%
Total Breads	209,886	100.00%	761	100.00%
Total Bakery Products	356,457.28		1354	

**Table 8:** Summary of the number of grain products for which a given feature is specified (based on public food procurements).

Criteria	Flours	Groats	Rice	Cereal Flakes	Bakery Products	Total
Product composition						
without additives	7	7	12	0	237	263
sugar/sweeteners	5	7	0	74	10	96
sodium/salt	5	7	0	25	0	37
characteristics typical of the products	148	85	115	115	359	822
without pollution	36	64	46	50	0	196
no gluten	96	0	0	12	17	125
without any criteria	335	692	259	452	942	2680
Sensorial characteristics						
taste	0	5	0	21	0	26
smell	3	13	9	22	61	108
color	32	25	16	12	60	145
appearance/consistency	18	127	21	73	194	433
without any criteria	496	977	315	494	1124	3406
Sustainable public procurements						
locality of products	4	0	0	0	0	4
organic products	0	0	0	0	6	6
packing method (cartonboard/glass)	58	19	13	5	3	98
big packing volume (>1 kg)	3	44	12	6	84	149
without any criteria	470	973	353	557	1265	3618

## Data Availability

Dataset available on request from the authors.
